# Genetic and environmental contributions to gaze lateralization across social and non-social stimuli in human infants

**DOI:** 10.1038/s41598-024-54373-6

**Published:** 2024-02-14

**Authors:** Charlotte Viktorsson, Ana Maria Portugal, Terje Falck-Ytter

**Affiliations:** 1 Development and Neurodiversity Lab, Department of Psychology, Uppsala University, Uppsala, Sweden; 2 Division of Neuropsychiatry, Department of Women’s and Children’s Health, Center of Neurodevelopmental Disorders (KIND), Karolinska Institutet, Stockholm, Sweden

**Keywords:** Behavioural genetics, Human behaviour

## Abstract

A tendency to look at the left side of faces from the observer’s point of view has been found in older children and adults, but it is not known when this face-specific left gaze bias develops and what factors may influence individual differences in gaze lateralization. Therefore, the aims of this study were to estimate gaze lateralization during face observation and to more broadly estimate lateralization tendencies across a wider set of social and non-social stimuli, in early infancy. In addition, we aimed to estimate the influence of genetic and environmental factors on lateralization of gaze. We studied gaze lateralization in 592 5-month-old twins (282 females, 330 monozygotic twins) by recording their gaze while viewing faces and two other types of stimuli that consisted of either collections of dots (non-social stimuli) or faces interspersed with objects (mixed stimuli). A right gaze bias was found when viewing faces, and this measure was moderately heritable (A = 0.38, 95% CI 0.24; 0.50). A left gaze bias was observed in the non-social condition, while a right gaze bias was found in the mixed condition, suggesting that there is no general left gaze bias at this age. Genetic influence on individual differences in gaze lateralization was only found for the tendency to look at the right versus left side of faces, suggesting genetic specificity of lateralized gaze when viewing faces.

## Introduction

Cerebral lateralization refers to the functional specialization of the two hemispheres of the brain. This lateralization has been found in a large number of species from different ecosystems, suggesting that it provides an important fitness benefit^1^. In line with this idea, many studies have found evolutionary advantages associated with hemispheric lateralization (see^2^ for a review). In humans, functional lateralization has been found, for example, in perception^3^, motor function^4^, language^5^, and memory^6^, and many studies have found links to structural asymmetries in the brain^2^. Additionally, findings suggest some shared lateralization patterns between humans and some non-human animal species, such as a left hemisphere specialization for communication^7^ and a right-hemisphere bias for social responsiveness (e.g.^8,9^).

Looking at the face of others is an important aspect of social communication, because it gives us information about the intentions, emotional states, and familiarity of others. Several studies have found that humans have a tendency to preferentially look at the right side of faces (i.e., looking to the left side of the face from the observers’ point of view). This left gaze bias has been extensively studied in adults (e.g.^10–12^) and has been hypothesized to be a tendency based on a right hemisphere advantage in social perception and face processing^13^, given that visual information in the left hemifield is initially directed to the right hemisphere. In line with this idea, a left visual field advantage has been found in determining gender and facial expressions^10^, and gaze direction is more accurately determined when presented in the left, rather than the right, visual field^14^. In addition, right hemispheric activation has been found during processing of emotional facial expressions^15^, supporting the hypothesis of a right hemisphere advantage in face processing. Guo et al.^12^ found that the left gaze bias was consistent in adults across different facial expressions and different tasks, suggesting that it might be an automatic lateralization process independent of perceptual processing of specific facial information. Several theories have attempted to explain the right hemisphere dominance in face processing. It has been suggested that the right hemisphere attends to both the left and right hemifield, while the left hemisphere only attends to the right hemifield, resulting in left-sided neglect when the right hemisphere is damaged^16^. Others have suggested that the right hemisphere rely on configural processing, while the left hemisphere rely on featural processing (e.g.^17^). This view has been supported by findings showing a greater left-sided perceptual bias in emotion judgement tasks when viewing upright faces (configural processing) as compared to when viewing inverted faces (featural processing; e.g.^18^).

Despite the large number of studies analyzing the left gaze bias in adulthood, relatively few studies have focused on lateralization of gaze in childhood and infancy. However, a face-specific left gaze bias has been found in 4-year-olds^19^ and 5-year-olds^20^. One study found a left gaze bias when viewing faces already at 3 months of age, but only for the lower part of the face when viewing naturally moving face stimuli^21^. A face-specific left gaze bias has also been found at 11 months of age^22^, while two other studies found that infants at 6 and 14 months of age show a left gaze bias for both faces *and* objects^23,24^, suggesting that gaze lateralization in infancy might be general and develop into being face-specific over time, possibly through the process of perceptual narrowing. This means that the face-specific left gaze bias found in older children and adults might be an experience-dependent process of gradual specialization, where infants start out with a general left gaze tendency, which through the experience of looking at a vast amount of faces, develops into a face-specific left gaze bias. A similar gradual specialization has been proposed for face recognition^25,26^. However, it is not known when this potential transition from a general to a face-specific left gaze bias is happening, and how experience might shape this trajectory.

While studies of lateralization in typically developing children sheds light on the early emergence of cerebral asymmetries and the potential influence on social and cognitive abilities in typical development, it is also important to explore whether atypical patterns of lateralization are present in neurodevelopmental conditions. One such condition is autism, which is characterized by difficulties in social communication, restrictive/repetitive behaviors, and sensory atypicalities^27^. A growing body of literature has found atypical lateralization in autistic individuals, including atypical lateralization of motor circuit connectivity^28^, structural asymmetry in language regions^29^, and ambiguous hand preferences^29^. Several studies have shown that children and toddlers with autism process faces differently than typically developing children^30–32^, sparking the question of whether there might be differences in gaze lateralization between autistic and non-autistic children. Dundas et al.^22^ found that infants with an elevated likelihood of later being diagnosed with autism did not show a left gaze bias when viewing faces at 11 months of age, contrary to the typical-likelihood infants in the study. In another study using a face-pop-out task, 6-month-old infants who later received an autism diagnosis showed a preference for stimuli (both faces and objects) on the right side, while the infants who did not receive a diagnosis showed the expected general left gaze bias (for both faces and objects^24^). At 14 months, however, infants with later autism reached the same level of left gaze bias as the typically developing infants, and no longer showed a preference for looking at stimuli to the right. The authors suggest that this pattern could be due to a delay in development, or the emergence of compensatory mechanisms. Further research on the potential link between gaze lateralization and autism is therefore needed in order to elucidate the putative association and the importance for later development.

In this study, we analyzed the specificity of gaze lateralization in early infancy (5 months of age) by presenting both faces and broader classes of stimuli. The face-specific condition consisted of dynamically moving faces (social condition), while the other conditions consisted of collections of dots (non-social condition) and static faces interspersed with non-social objects (mixed condition). Based on earlier findings of a left gaze bias in infants for both social and non-social stimuli (e.g.^23^), we hypothesized that we would find, at group level, a significant preference for the left side of the face in the Social condition and the left side of the screen in the Non-social and Mixed conditions, and that the gaze lateralization in these conditions would be correlated.

While functional lateralization seems to be evolutionary advantageous^2^, the heritability of, for example, handedness and speech laterality in adulthood is low (Refs.^7,33^ respectively, but see also^34^ regarding hand preference), suggesting a large influence of environmental factors. This probes the question of whether gaze lateralization is influenced mainly by genetics or environments. In order to elucidate the etiology of gaze lateralization in early infancy and increase the understanding of the emerging left gaze bias, we analyzed the genetic and environmental (shared and unique) influence on individual differences in gaze lateralization, using the classical twin approach of comparing similarity in monozygotic and dizygotic twin pairs.

Due to the findings of early differences in gaze lateralization in children with and without later autism^22,24^, we also explored the potential links between gaze lateralization in infancy and socio-communicative abilities at 14 months and autistic traits at 36 months. Finally, it is largely unknown whether gaze lateralization in infancy is associated with other concurrent and later emerging abilities. Therefore, we explored potential links between gaze lateralization in infancy and concurrent developmental level as well as language comprehension at 14 months and vocabulary at 36 months. These analyses were pre-registered at OSF (https://osf.io/kpqfx/).

## Methods

### Participants

The sample consisted of participants in the longitudinal BabyTwins Study Sweden (BATSS^35^), which were recruited from the national population registry (only the greater Stockholm area was selected, due to in-person assessments in Stockholm). From 2016 to 2020, 311 families (29% of the entire population of same-sex twins born in the area) participated in the multi-methods assessment at 5 months. Data collection was performed at the Centre of Neurodevelopmental Disorders at Karolinska Institutet (KIND) in Stockholm, Sweden. In general, the study sample has a high socioeconomic status, and it includes mainly Swedish-born families (90% of twin pairs had at least one parent born in Sweden). See Table 1 for sample demographics (in-depth demographics are reported elsewhere^35^). Parents gave informed consent to take part. BATSS was approved by the regional ethics board in Stockholm and was conducted in accordance with the Declaration of Helsinki.

**Table 1:** Descriptive statistics.

	Mean (SD)^a^ [min; max]		
	Total (n = 592)	MZ (n = 330)	DZ (n = 262)
N females (%)	282 (47.6%)	152 (46.1%)	130 (49.6%)
Age (in days)^b^	167.5 (8.8) [145.0; 203.0]	167.5 (8.6) [149.0; 194.0]	167.6 (8.9) [145.0; 203.0]
Parental education^c^	3.30 (0.74) [1.50; 4.00]	3.27 (0.76) [1.50; 4.00]	3.33 (0.71) [1.50; 4.00]
Family income^d^	6.58 (2.35) [1.00; 10.00]	6.50 (2.28) [1.00; 10.00]	6.69 (2.43) [1.00; 10.00]

^a^Except for N females, which shows the frequency.

^b^4 twin pairs differed in age, in these cases the mean age was used.

^c^Education level on a scale from 1 to 4, where 1 = Primary, 2 = Secondary, 3 = Undergraduate (≤ 3 years) and 4 = Postgraduate level (> 3 years).

^d^Family income per month. Scale 1–10 where 1 =  < 20 K, 2 = 20–30 K, 3 = 30–40 K, 4 = 40–50 K, 5 = 50–60 K, 6 = 60–70 K, 7 = 70–80 K, 8 = 80–90 K, 9 = 90–100 K and 10 =  > 100 K (SEK).

General exclusion criteria for the study were opposite-sex twin pairs, diagnosis of epilepsy, known presence of genetic syndrome related to autism, uncorrected vision or hearing impairment, very premature birth (prior to week 34), presence of developmental or medical condition likely to affect brain development (e.g., Cerebral Palsy), and infants where none of the biological parents were involved in the infant’s care. Among the recruited and tested infants, 3 twins were excluded from analysis because they subsequently were found not to fulfil the general criteria (above) due to seizures at the time of birth (n = 2 infants) and spina bifida (n = 1 infants). In addition, we excluded 24 infants due to twin-to-twin transfusion syndrome (12 twin pairs) and one infant due to birthweight below 1.5 kg. Condition-specific criteria for exclusion are described in the “Eye tracking procedure and stimuli” section.

### Eye tracking procedure and stimuli

The stimuli used in this study (Non-social condition^36^; Social condition^37^; Mixed condition^38^) were not purposefully designed to measure gaze lateralization, but were used for this purpose as we believe they fulfil the characteristics necessary to answer our research questions. In all three conditions, the sample is based on the same set of infants from BATSS^35^, although the sample varies slightly in each condition due to stimuli-specific exclusion criteria (see specifications in each condition section).

For the Social and Non-social conditions, gaze data was recorded using the Tobii T120 eye-tracker with a sampling rate of 60 Hz, using a standard Tobii monitor at native resolution (1024 × 768). For the mixed condition, a Tobii TX300 eye-tracker was used, with a sampling rate of 120 Hz. The infant was seated in a baby chair or in the parent’s lap, approximately 60 cm from the screen. Before the eye tracking session, a 5-point calibration video was presented, and the experimental task did not begin until a successful calibration was achieved. For the Social and Non-social conditions, another 5-point video for offline calibration validation purposes was shown once in the beginning of the eye-tracking session.

#### Social condition

**Figure 1:**
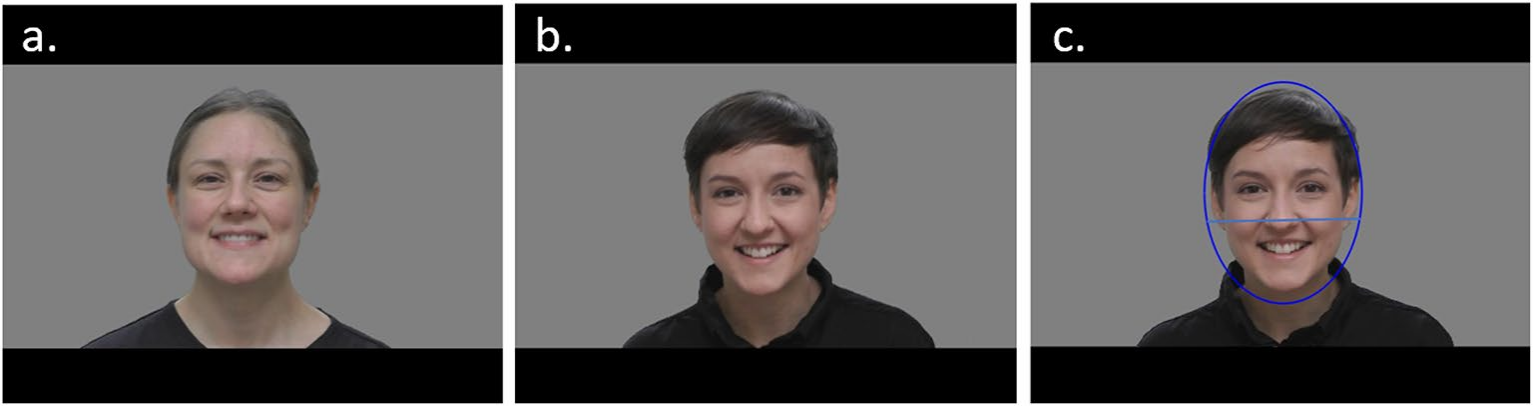
Experimental stimuli from the Social condition. (**a,b**) The videos comprised a set of face stimuli (still, speaking, singing) with the natural voice sound included, from two different models. (**c**) The face AOI used for analysis, divided in an upper and a lower part. Figure is adapted from Viktorsson et al.^37^, and used with permission from authors.

The stimuli consisted of 12 videos in which a woman sings nursery rhymes, 4 videos in which a woman is talking (rhyme verses) and 4 videos in which a woman is only smiling (Fig. 1). The primary goal of these stimuli was to measure eye versus mouth looking (already published data^37^). The videos were shown in a pseudo-random order, unique to each participant. In all videos, a woman was centered in the video and the background was grey (there were two women, each of them contributing equally to all conditions). The length of the videos ranged from 4 to 12 s (total duration was 153 s). Further details are provided elsewhere^37^. A dynamic area of interest (AOI) was created for each frame of the videos (Fig. 1c). The horizontal radius of the ellipse is 200 pixels, and the vertical radius is 280 pixels. In an earlier study of the tendency to look at the eyes and the mouth using the same stimuli and sample^37^, it was found that most infants tended to look at the eyes. Since the eyes are also spatially separated from each other, the face AOI was divided into an upper and a lower part (Fig. 1c), and in a deviation from the analysis plan, the eye region was primarily used for all further analyses (analyses involving the mouth region are reported in Supplementary Information S1). Lateralization was measured as the total amount of time looking at the left hemiface relative to the total amount of time looking at the whole face, calculated separately for the lower and the upper part of the face (giving us a scale of 0–1, where 0 means not looking at the left hemiface at all, while 1 means looking at the left hemiface 100% of the time). A value over 0.5 means, therefore, that there is a left gaze bias, while a value below 0.5 suggests a right gaze bias. Inclusion criteria for this condition was looking at the face for at least 20% of the total duration of the condition (i.e., 30.6 s; exclusions were not made on a trial-by-trial basis, since the stimuli was very similar in all videos and always centered in the video). In total, 21 participants were excluded from this condition due to this criterion. In addition, 2 infants were excluded due to non-Swedish speaking parents, 6 due to technical issues, 7 due to lack of time, 2 due to lack of room, and 4 due to being too tired or too fussy. The final sample in this condition consisted of 552 infants.

#### Non-social condition

**Figure 2:**
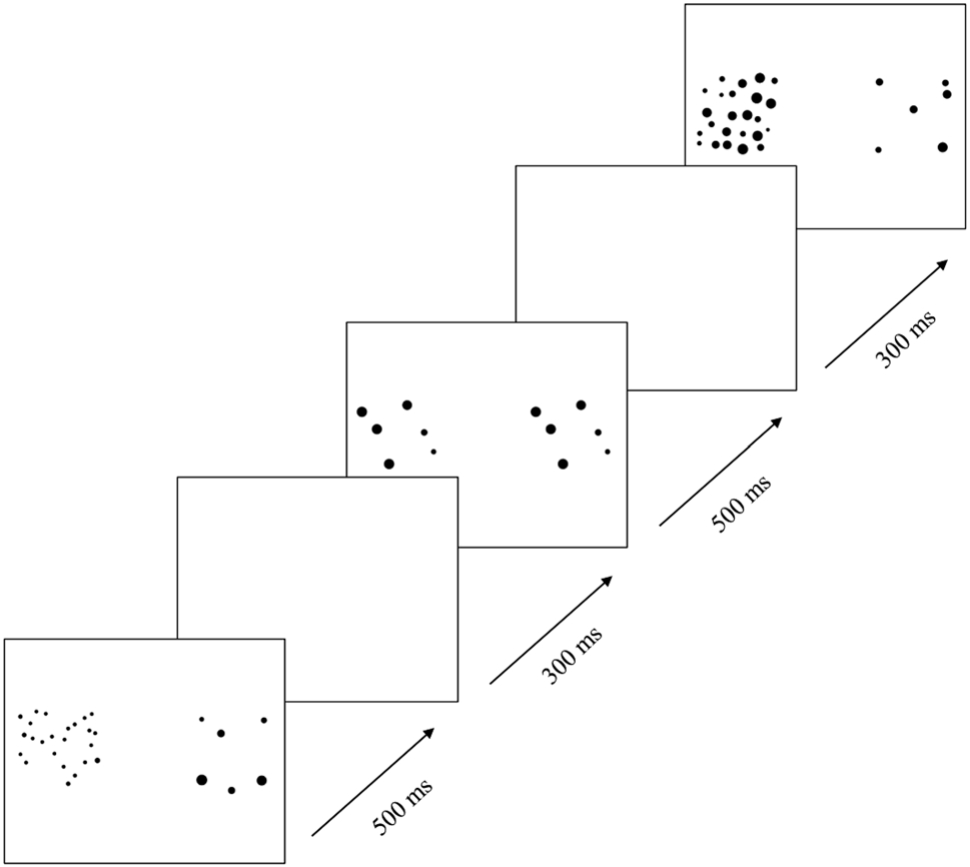
Experimental stimuli from the non-social condition. each image consisted of two sets of dots that was presented for 500 ms, followed by a blank screen for 300 ms. Every other image showed identical sets of dots on the right and left side the screen while remaining images differed in numerosity on the two sides. Reproduced with permission from authors^36^.

The stimuli consisted of 8 videos (each shown for 16 s), which contained a series of images, each of which showed two sets of dots, appearing on the left and right sides of the screen (Fig. 2). The primary goal of these stimuli was to measure the approximate number system (already published data^36^). Each image was unique in terms of a specific spatial constellation of dots. On one side of the screen, the collection of dots was numerically constant, while on the other side the collection of dots alternated in numerosity. The side with alternating numerosity switched between 10 and 20 dots (1:2 ratio condition) or 6 and 24 dots (1:4 ratio condition). The side with constant set sizes showed 10 dots and 6 dots, respectively, for these conditions. Each condition consisted of four stimulus videos, which were counterbalanced in terms of left vs. right location of the side with alternating set size. In half of the images where the two sets of dots differed in numerosity, the two sets of dots were matched on the total surface area. In the other half, the two sets of dots were matched on individual dot size. In 50% of the videos, the two sets of dots were controlled for convex hull (the smallest convex polygon that contains a set of dots). Lateralization in this condition was measured as the amount of time looking at the left side of the screen, relative to the amount of time looking at the whole screen. In order to create this variable, we averaged the percentage of viewing time at the left side of the screen (relative to the whole screen) for trials where the numerically changing side was on the left side and on the right side, respectively. This was done separately for each condition, and was then averaged to create the final variable (there was no statistically significant difference in lateralization between the two conditions; t(513) =  − 0.830, p = 0.407). By doing this, the final measure contained the same amount of information from trials where the numerically changing side was on the left side of the screen as from trials where the opposite was true, creating a non-biased variable. Inclusion criteria for this condition was looking at the screen for at least 20% of the total duration of each video (approximately 3.2 s), in order to allow the infants to observe the numerically changing dots. Infants were included in further analyses only if they had at least four valid trials (of which two from each condition, counterbalanced in terms of left vs right location of the numerically changing side). Due to these criteria, 61 participants were excluded from this condition. In addition, 6 infants were excluded due to technical issues, 7 due to lack of time, 2 due to lack of room, and 4 due to being too tired or too fussy. The final sample in this condition consisted of 514 infants.

#### Mixed condition

**Figure 3:**
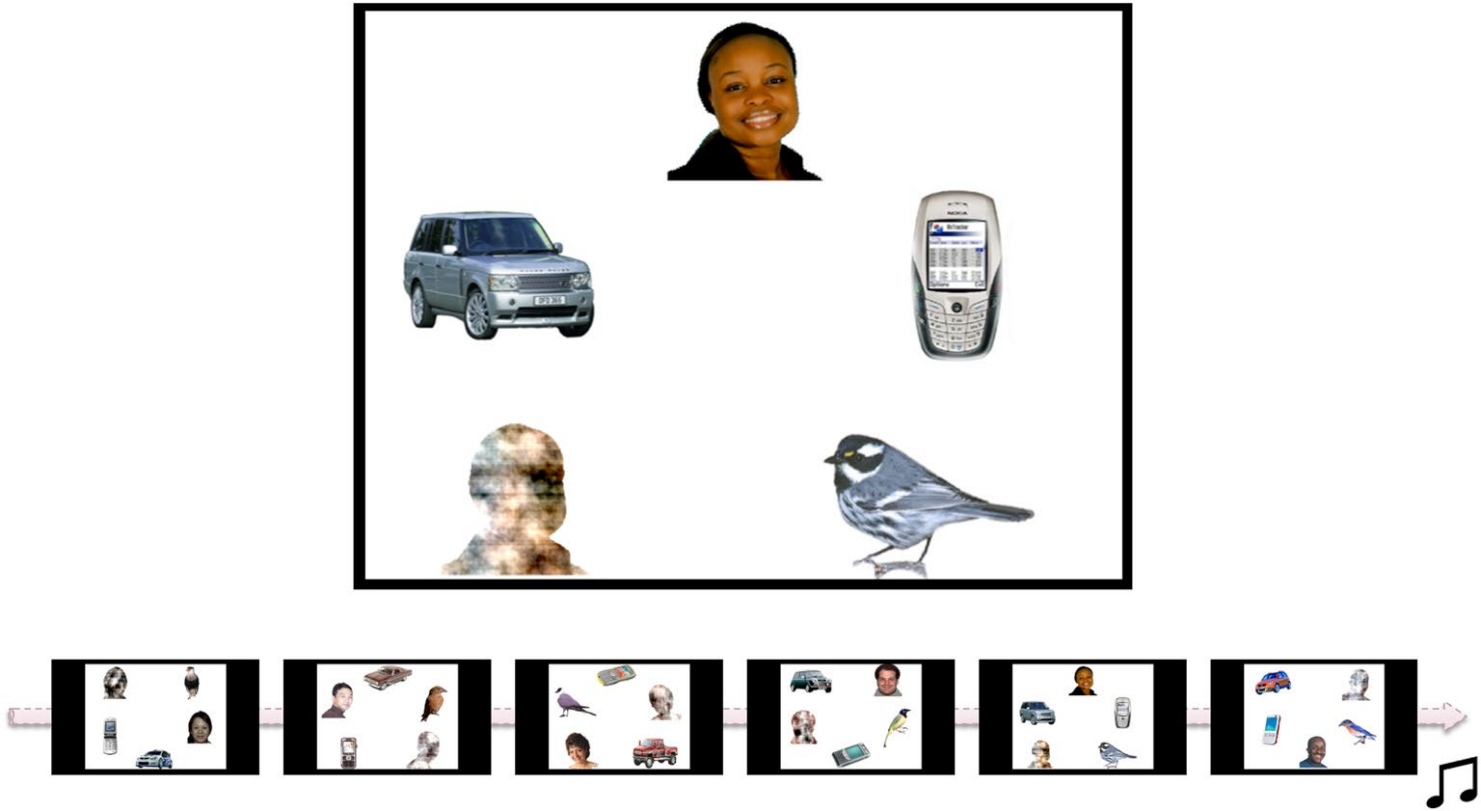
Experimental stimuli from the mixed condition. Each image consisted of five pictures, of which one was a face and one was a noise stimulus made from the face. The images were presented in a fixed order shown in the scheme. Only data from the first four images were used in this study (where the face is either to the left or to the right). Reproduced with permission from authors^38^.

Stimuli consisted of 6 different complex displays of objects (Fig. 3), including a face (with direct eye-gaze; counterbalancing ethnicity and location of the face within the array) and 4 non-face competitors (including a “noise” stimulus generated from the same face, a mobile phone, a bird, and a car). The primary goal of these stimuli was to measure face orienting and preference (already published data^38^). In two trials the face was to the right of the screen, in two trials it was to the left, and in two trials the face was in the middle of the screen either at the top (array in Fig. 3) or at the bottom of the screen. In a deviation from the pre-registered analysis plan, we only included the four trials where the face was either to the left or to the right of the screen, since it was found in an earlier study that the infants preferred looking at the face when viewing these images^38^ and the lateralization measure therefore might be biased if images with faces in the middle of the screen are included. These images were shown for 20 s each, in a fixed order. Lateralization in this condition was measured as the amount of looking time at the left side of the screen, relative to the whole screen (first averaged for valid trials where the face was either to the left or to the right, to create an unbiased average lateralization score). A value over 0.5 means, therefore, that there is a left gaze bias, while a value below 0.5 suggests a right gaze bias. A trial was classified as valid if the infant looked at the screen for at least 20% of the total duration of the video (i.e., 4 s, as the total duration was 20 s). Infants were included in further analyses only if they had at least two valid trials, of which one where the face was to the left and one where the face was to the right. Due to this criterion, 14 participants were excluded from this condition. In addition, 23 participants did not partake in this experiment due to lack of time or technical issues, and were therefore not included in this condition. The final sample in this condition consisted of 559 infants.

### Parent-rated questionnaires

The CSBS DP Infant Toddler Checklist, ITC^39^, is a 24-item parent-rated questionnaire, used to identify children with any type of communication delay. Lower scores indicate a higher degree of socio-communicative delays. Items include, for example, questions on whether the parent knows when the child is happy or sad, and whether the child lets the parent know when they need help reaching an object. It was administered at 14 months (age range was 387–525 days), and we used the total score as a measure of socio-communicative behaviors. Data from one individual was excluded due to too old age (806 days).

The MacArthur Communicative Development Inventory, CDI^40^, is a parent-rated questionnaire that assesses early language development. It was administered at 14 months (the Words and Gestures form, age range was 386–516 days) and 36 months (the Words and Sentences form, age range was 1086–1401 days). As a measure of receptive vocabulary at 14 months, we used the total number of words (out of 370 words) that the infant could understand but not produce. At 36 months, we used the vocabulary checklist score as a measure of expressive vocabulary.

The Quantitative Checklist for Autism in Toddlers, Q-CHAT^41^, is a normally distributed quantitative measure of autistic traits, which consists of 25 parent-rated items scored on a 5-point scale (0–4) and was administered at 36 months (age range was 1074–1401 days). The scores from all items are summed to obtain a total score, where higher scores indicate more autistic traits. Data from two individuals were excluded due to insufficient age (735 days and 783 days).

### Experimenter-rated developmental assessment

The Mullen Scales of Early Learning, MSEL^42^, was administered by an experimenter at 5 months. This is a standardized assessment commonly used in many areas of psychology as a measure of general cognitive ability. Here, the Early Learning Composite Score was used (a standardized score derived from fine motor, visual reception, receptive language, and expressive language subscales).

See Table 2 for descriptive statistics on parent-rated questionnaires and the experimenter-rated developmental assessment.

**Table 2:** Descriptive statistics for concurrently and subsequently measured traits.

	Number of infants	Mean (SD) [min; max]	Skewness	Kurtosis
MSEL 5 months (ELCS)				
Females	270	95.39 (10.13) [67; 129]	0.64	0.74
Males	291	94.13 (8.73) [72; 132]	0.78	2.36
Total	561	94.73 (9.44) [67; 132]	0.73	1.43
CDI 14 months (comprehension)				
Females	195			
Males	211	75.68 (67.97) [3; 332]	1.48	2.08
Total	406	84.72 (68.17) [1; 382]	1.33	2.10
ITC 14 months				
Females	194	35.93 (6.30) [20; 52]	− 0.12	0.11
Males	211	34.06 (7.77) [11; 51]	− 0.35	− 0.17
Total	405	34.96 (7.16) [11; 52]	− 0.35	0.11
CDI 36 months (production)				
Females	194	567.99 (119.25) [137; 710]	− 1.41	2.24
Males	197	509.61 (166.95) [40; 710]	− 0.83	
Total	391	538.58 (147.98) [40; 710]	− 1.14	0.75
Q-CHAT 36 months				
Females	193	20.31 (7.25) [1; 41]	− 0.25	0.21
Males	197	23.30 (8.04) [3; 47]	0.26	0.15
Total	390	21.82 (7.80) [1; 46]	0.10	0.36

MSEL = Mullen Scales of Early Learning; CDI = Communicative Development Inventory; ITC = Infant-Toddler Checklist; Q-CHAT = Quantitative Checklist for Autism in Toddlers.

### Statistical analyses

Left versus right gaze bias was analyzed using one-sample t-tests comparing the mean lateralization against a value of 0.5 (i.e., chance level). Associations among eye tracking measures were analyzed using two-tailed Pearson correlations.

Univariate twin models were used to estimate the genetic and environmental contribution to the mean gaze lateralization in each condition. The sources of variation in a trait can be divided into genetic influences (A; heritability), shared environment (C; e.g., family environment), and unique environment (E; i.e., environmental influences that makes twins different from each other, including measurement error). Since monozygotic (MZ) twins share 100% of their segregating DNA, while dizygotic (DZ) twins on average share 50% of their segregating DNA, a higher within pair similarity among MZ twins than DZ twins suggests genetic contribution to a trait. Zygosity was determined for all twin pairs by DNA analysis. Sex and age were included as covariates in all twin models. The best fitting model was selected based on the Akaike Information Criterion (AIC).

All phenotypic associations were calculated using the robust sandwich estimator in generalized estimating equations (GEE) in order to account for the correlation between twins in a pair^43^, using the drgee package in R^44^. The variables used in these models were regressed on age and sex before analyses. Due to the explorative nature of the phenotypic analyses, we adjusted the p values using Bonferroni correction. The original significance threshold was p < 0.05 and the number of analyses was 15, meaning that the adjusted significance threshold was p < 0.003.

## Results

**Figure 4:**
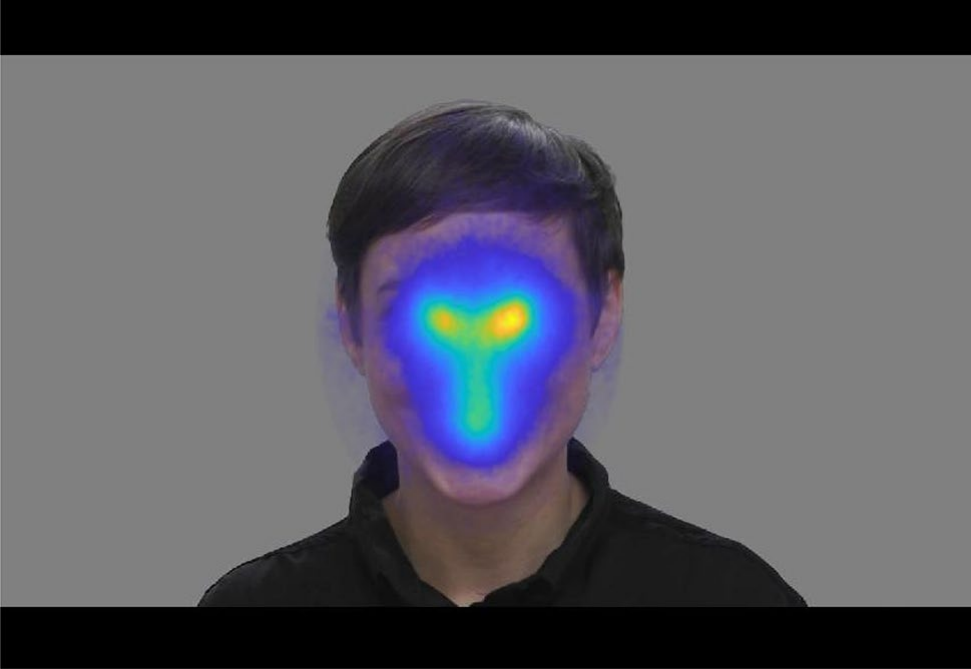
An aggregated heatmap of all gaze data (from all participants) inside the face AOI in the Social condition (adjusted for movement of the face).

Contrary to the pre-registered hypothesis, there was a statistically significant *right* gaze bias in the Social condition (t(551) =  − 11.63, p < 0.001, Cohen’s d =  − 0.50; see Fig. 4). A significant right gaze bias was also found in the Mixed condition (t(558) =  − 4.57, p < 0.001, Cohen’s d =  − 0.19), while a significant *left* gaze bias was found in the Non-social condition (t(513) = 68.08, p < 0.001, Cohen’s d = 3.00; Table 3). There was no significant difference in these results when analyzing twin 1 and twin 2 separately. There were clear individual differences in all conditions, suggesting that some individuals preferred looking at the left side and some preferred looking at the right side (Supplementary Information Fig. S1). In order to examine the stability of gaze lateralization throughout the trials, we split each trial in each condition, calculating the gaze lateralization for the first and second half of each trial (i.e., each video or image). We then estimated the mean gaze lateralization in each condition, separately for the first versus second half of trials, and compared these means using a paired-samples t-test. These analyses were not pre-registered. No statistically significant difference was found between the lateralization score in the first half versus the second half of the trials in the Mixed condition (mean first half = 0.466, mean second half = 0.475; t(479) =  − 1.302, p = 0.193) or in the Non-social condition (mean first half = 0.557, mean second half = 0.552; t(513) = 0.949, p = 0.343). In the Social condition, a statistically significant difference between the first half and the second half of the trials was found, but the mean lateralization scores were very similar and both indicated a right gaze bias (mean first half = 0.398, mean second half = 0.386; t(553) = 2.612, p = 0.009). As the right gaze bias in the mixed condition might reflect a tendency to look more at the non-social objects on the right side rather than the face, we conducted a follow-up (not pre-registered) analysis of the ratio of looking at the face related to the rest of the objects on the screen. The mean ratio of looking at the face was 0.36 when presented on the left side of the screen and 0.50 when presented on the right side of the screen, suggesting a stronger tendency to look at the face when presented on the right side. This difference was statistically significant (t(558) =  − 13.45, p < 0.001).

**Table 3:** Descriptives of eye tracking measures.

	N	Total	Mean (SD) [min; max]			
			MZ males	MZ females	DZ males	DZ females
**Social condition**	552					
Total looking time at screen (in seconds)		119.0 (30.0) [32.4; 162.3]	115.9 (32.0) [32.4; 160.4]	115.0 (32.2) [39.0; 160.7]	122.0 (26.1) [57.2; 159.6]	124.7 (27.2) [34.4; 162.3]
Mean lateralization^b^		0.390 (0.221) [0.001; 0.995]	0.389 (0.224) [0.009; 0.995]	0.376 (0.212) [0.002; 0.881]	0.407 (0.218) [0.003; 0.983]	0.393 (0.231) [0.001; 0.950]
**Non-social condition**	514					
Mean looking time at screen (in seconds)^a^		10.81 (2.26) [5.62; 15.71]	10.80 (2.27) [6.02; 15.25]	10.98 (2.31) [5.95; 15.65]	10.67 (2.28) [5.90; 15.71]	10.79 (2.20) [5.62; 15.28]
Mean lateralization^b^		0.554 (0.183) [0.026; 0.998]	0.550 (0.170) [0.026; 0.903]	0.559 (0.178) [0.191; 0.925]	0.548 (0.193) [0.101; 0.980]	0.560 (0.196) [0.101; 0.998]
**Mixed condition**	559					
Mean looking time at screen (in seconds)^a^		14.45 (3.13) [5.91; 20.19]	14.59 (3.02) [6.97; 20.04]	14.26 (3.19) [5.91; 19.71]	14.57 (3.18) [6.82; 20.19]	14.37 (3.16) [7.09; 19.87]
Mean lateralization^b^		0.472 (0.137) [0.151; 0.967]	0.481 (0.138) [0.170; 0.861]	0.461 (0.147) [0.151; 0.967]	0.473 (0.128) [0.174; 0.807]	0.472 (0.133) [0.170; 0.832]

^a^Averaged across all valid trials.

^b^Scale 0–1, where 0 = looking at the left side 100% of the time, 1 = looking at the right side 100% of the time.

Gaze lateralization was not associated with total looking time at the screen in the Social condition (r =  − 0.019, p = 0.657), and not with mean looking time at the screen (averaged across valid trials) in the Non-social condition (r = 0.039, p = 0.378), or Mixed condition (r = 0.056, p = 0.190). Gaze lateralization in the Social condition was not associated to lateralization in the Mixed condition (r =  − 0.001, p = 0.988), but it was significantly associated with the gaze lateralization in the Non-Social condition (r = 0.100, p = 0.026), meaning that infants who looked to the left when viewing faces also tended to look to the left when viewing collections of dots. There was also a statistically significant association between the Non-social condition and the Mixed condition (r = 0.143, p = 0.002).

### Genetic analyses

The twin correlations for the Social condition suggested genetic influence on this measure (i.e., MZ correlation higher than DZ correlation), but the twin correlations for the Non-social and Mixed conditions did not (Table 4). The lateralization score for all conditions met the assumptions for twin modelling (equal variances and means across twin and zygosity), see Supplementary Information Table S1.

**Table 4:** MZ and DZ intra-class correlations for mean lateralization in each condition.

	MZ ICC (95% CI)	DZ ICC (95% CI)
Social condition	0.37 (0.30; 0.43)	0.15 (− 0.04; 0.33)
Non-social condition	0.10 (− 0.08; 0.28)	0.21 (0.03; 0.37)
Mixed condition	0.07 (− 0.09; 0.23)	0.07 (-0.11; 0.25)

Next, an ACE model was fitted, separately for each condition (Table 5). The best fitting model for the Social condition was an AE model, where the shared environment component was dropped. This model suggested moderate genetic influence on lateralization in the Social condition (A = 0.38, 95% CI 0.24; 0.50, E = 0.62, 95% CI 0.50; 0.76). The best fitting model for the Non-social condition was a CE model, which suggested a low influence of shared environment and a large influence of unique environment (C = 0.16, 95% CI 0.03; 0.28, E = 0.84, 95% CI 0.72; 0.97). The best fitting model for the Mixed condition was an E model, meaning that no familial factors (genetic or non-genetic) influences the gaze lateralization in this condition (E = 1.00).

**Table 5:** Univariate twin models for the Social, Non-social, and Mixed condition.

Model	− 2LL	#parameters	*df*	AIC	Comparison model	Δ χ^2^	Δ df	p	A	C	E
Social condition											
Fully sat.	− 132.86	12	538	− 108.86	–	–	–	–	–	–	–
ACE	− 126.38	6	544	− 114.38	Fully sat.	6.48	6	0.37	0.38	< 0.001	0.62
**AE**	− **126.38**	**5**	**545**	− **116.38**	**ACE**	** < 0.001**	**1**	**1.00**	**0.38**	–	**0.62**
CE	− 121.71	5	545	− 111.71	ACE	4.67	1	0.03	–	0.28	0.72
E	− 101.11	4	546	− 93.11	ACE	25.27	2	< 0.001	–	–	1.00
Non-social condition											
Fully sat.	4415.20	12	500	4439.20	–	–	–	–	–	–	–
ACE	4421.78	6	506	4433.78	Fully sat.	6.57	6	0.362	< 0.001	0.16	0.84
AE	4423.29	5	507	4433.29	ACE	1.51	1	0.218	0.18	–	0.82
**CE**	**4421.78**	**5**	**507**	**4431.78**	**ACE**	** < 0.001**	**1**	**1.00**	–	**0.16**	**0.84**
E	4427.80	4	508	4435.80	ACE	6.02	2	0.049	–	–	1.00
Mixed condition											
Fully sat.	− 645.13	12	545	− 621.13	–	–	–	–	–	–	–
ACE	− 640.91	6	551	− 628.91	Fully sat.	4.23	6	0.646	< 0.001	0.07	0.92
AE	− 640.73	5	552	− 630.73	ACE	0.18	1	0.674	0.08	–	0.92
CE	− 640.91	5	552	− 630.91	ACE	< 0.001	1	1.00	–	0.07	0.93
**E**	− **639.50**	**4**	**553**	− **631.50**	**ACE**	**1.40**	**2**	**0.495**	–	–	**1.00**

The best fitting model for each condition is marked in bold.

### Phenotypic analyses

After correcting for multiple testing, there were no statistically significant associations between the lateralization in any of the conditions at 5 months and concurrent general development, socio-communicative abilities at 14 months, or language comprehension at 14 months. Likewise, autistic traits and vocabulary at 36 months were not associated with gaze lateralization in any of the conditions (see Table 6 for details).

**Table 6:** GEE analyses of the association between lateralization scores and phenotypic measures.

	Social condition		Non-social condition		Mixed condition	
	β (95% CI)	p	β (95% CI)	p	β (95% CI)	p
MSEL 5 months (ELCS)	0.02 (− 0.06; 0.11)	0.551	0.07 (− 0.02; 0.16)	0.110	− 0.09 (− 0.18; < − 0.01)	0.043
CDI 14 months (comprehension)	− 0.01 (− 0.12; 0.10)	0.846	− 0.08 (− 0.18; 0.02)	0.121	− 0.01 (− 0.13; 0.11)	0.861
ITC 14 months	0.01 (− 0.11; 0.12)	0.913	− 0.11 (− 0.21; < − 0.01)	0.040	− 0.06 (− 0.17; 0.06)	0.328
CDI 36 months (production)	− 0.04 (− 0.16; 0.09)	0.535	− 0.01 (− 0.13; 0.12)	0.885	− 0.01 (− 0.11; 0.09)	0.796
Q-CHAT 36 months	− 0.08 (− 0.18; 0.03)	0.155	− 0.04 (− 0.15; 0.07)	0.480	− 0.06 (− 0.16; 0.04)	0.264

MSEL = Mullen Scales of Early Learning; CDI = Communicative Development Inventory; ITC = Infant-Toddler Checklist; Q-CHAT = Quantitative Checklist for Autism in Toddlers.

## Discussion

The aims of this study were to estimate gaze lateralization during face observation and to more broadly estimate lateralization tendencies across a wider set of stimuli, as well as estimating the influence of genetic and environmental factors on lateralization of gaze. Adults tend to look to the left when viewing faces (e.g.^12^), and some studies have found a similar left gaze bias in infancy (e.g.^22^). Here, we did not find a left gaze bias when presenting faces (either alone or interspersed with non-social objects), but instead a significant right gaze bias. Although we did find a left gaze bias when the infants were viewing non-social events (collection of numerically changing dots), these results suggest that there is no general or face-specific left gaze bias at this age. While Xiao et al.^21^ found a left-side preference when viewing faces already at 3 months of age, they only found this preference for the lower half of the face, which is notable considering that infants at that age mainly look at the eyes^45^. Furthermore, they did not find a left-side preference for the same face flipped horizontally, even when only analyzing the lower half of the face^21^. Guo et al.^23^ found a left gaze bias at 6 months of age for both faces and non-social objects, but their sample size was noticeably small (N = 19). Our findings, in a sample of 592 infants, do not support the idea of either a general or a face-specific left gaze bias, suggesting that the left gaze bias found in older children and adults is not yet present at five months of age.

What may be the cause of the right gaze bias for faces in early infancy? At 5 months of age, the brain is not yet mature, and myelination is on-going^46^, meaning that a specialized circuitry for face processing might not yet have emerged. It has been argued that the neural specialization for face processing is a product of an innate preference for orienting towards faces, and the experience gained from looking at a vast amount of faces during development^47^. Therefore, it is plausible that infants in the first few months of life have not yet gained enough experience with faces to develop certain specialized cortical circuits involved in face processing. That may explain why we do not find a *left* gaze bias, which is often attributed to a right hemisphere advantage in face processing^13^. In the absence of a holistic face processing module, it is possible that the left hemisphere specialization in fine-grained processing^48^ is driving the gaze to the right. However, more research is needed in order to understand the emergence of the left gaze bias later in childhood, and what may influence this shift in gaze bias from the right (in infancy) to the left (later in development) when looking at faces^19,20^. It is important to note that there were considerable individual differences in gaze lateralization in our sample, and another important goal of future research is understanding how these individual differences develop throughout childhood.

Individual differences in the tendency to look at the left or the right side of faces were moderately heritable (*h*^2^ = 0.38), which, although speculative, might reflect differences in the timing of transition to a stable face-specific left gaze bias. Other aspects of face perception have been found to be heritable in early infancy, such as face preference in the face-pop-out task (*h*^2^ = 0.46^38^) and the preference for looking at eyes versus mouth (*h*^2^ = 0.57^37^). These results support the idea that, already from a very early age, faces are special, and individual differences in face processing are partly due to genetics. Individual differences in gaze lateralization for mixed and non-social stimuli did not show any influence of genetics. Instead, shared environment had a modest influence on gaze lateralization in the non-social condition, while most of the variance was due to unique environment. Familial factors (genetics and shared environment) did not influence gaze lateralization in the mixed condition. This supports the idea that different etiologies may underlie lateralized looking at faces versus other stimuli. While the mixed condition consisted of stimuli that included faces, these images were static and the faces were interspersed with other non-social objects. In contrast, the stimuli in the social condition consisted of dynamic, naturally moving faces in the center of the screen, without any distractors. An earlier study from our lab using the same stimuli as in the mixed condition (faces interspersed with objects) showed that the infants tended to look more at the face than the other objects^38^, meaning that the lateralization measure in this condition tells us whether lateralization of gaze goes above and beyond the general preference for faces. As the placement of the face to the right versus the left is balanced in our design, a lateralization score below 0.50 suggests that there is a general tendency (on a group level) to look slightly more at the face when it is presented to the right. This was supported by the ratio of looking at the face (versus the rest of the objects) being significantly higher when the face was presented to the right, as compared to when presented to the left. The lack of association between the gaze lateralization in the social and mixed conditions suggests that different mechanisms underlie the tendency to look at the left or right side of faces as compared to orienting towards faces that are placed to the left or to the right on the screen.

In the current study, a left gaze bias was found (on group level) in the non-social condition, where the infants watched videos of numerically changing collection of dots. This might suggest that in early infancy, there is a left gaze bias for non-social stimuli only. It is also possible that the left gaze bias for non-social stimuli found in our study is stimulus-specific and related to numerical sense, as several studies have found that humans represent number magnitude on a left-to-right oriented number line^49^ and it recently has been found that newborns show a left visual preference when processing numerical quantity^50^. It has also been found that both nutcrackers and domestic chicks show a leftward bias when locating an object based on its ordinal position^51^, suggesting an evolutionary base for the disposition to map numbers from the left to the right. However, the mean lateralization score was only slightly higher than chance level (0.55), and there were considerable individual differences (Supplementary Information Fig. S1). It is therefore plausible that this weak experimental effect reflects chance factors in this large sample. The same can be said about the right gaze bias in the mixed condition, which, although statistically significant, was very close to chance level (0.47). The significant association between the gaze lateralization in the non-social and mixed condition suggests that infants had a general tendency to look at the right or the left side, regardless of whether they viewed collections of dots or faces interspersed with objects. However, as the mean values were close to chance level, it is important to interpret these findings cautiously. It is notable, though, that we did find statistically significant biases in gaze lateralization using videos and images where we corrected for potential lateral differences in the stimuli. This suggests that lateralized biases might be a problem in infant eye tracking, indicating that gaze lateralization should be analyzed in studies where gaze towards stimuli presented on either side of the screen is important for the research question.

No associations were found between gaze lateralization in any condition and socio-communicative abilities at 14 months or autistic traits at 36 months. Although it has been found that infants with an elevated likelihood of autism show a weaker left gaze bias than typically developing infants at 6 and 11 months, respectively^22,24^, this difference is no longer evident in 14-month-olds^24^. The lack of association between gaze lateralization and autistic traits in our study might reflect the fact that our sample consisted of typically developing infants, who generally did not display high levels of autistic traits later in development. Alternatively, the association might not be evident already at 5 months of age. Further studies are needed in order to elucidate the potential relationship between gaze lateralization and autistic traits in both typically developing children and children with later autism.

We did not find any statistically significant associations between gaze lateralization in any of the conditions at 5 months and concurrent general development. Likewise, no association was found with language comprehension at 14 months or vocabulary at 36 months. This suggests that the tendency to look at the left or the right side of both social and non-social stimuli in early infancy is not related to development in toddlerhood. Future studies should focus on analyzing the developmental trajectory of the face-specific left gaze bias that emerges later in life, and the potential importance for cognitive and social abilities throughout childhood.

### Limitations

A limitation of the current study is that the stimuli used was not purposefully designed to measure gaze lateralization. Therefore, the comparison between conditions is not optimal. For example, in order to compare lateralization in social and non-social conditions, it would have been more appropriate to use objects the same size as faces, presented in the middle of the screen. Instead, we had to rely on the looking time at left versus right side of the screen in a paradigm designed to measure the approximate number system.

## Conclusions

At 5 months of age, infants show a left gaze bias when viewing non-social stimuli (collection of numerically changing dots), and a right gaze bias when viewing dynamic faces and static faces interspersed with non-social objects. Individual differences in the tendency to look at the left or the right side of faces were moderately heritable, while no genetic influence was found for gaze lateralization when looking at more broad classes of social and non-social stimuli, suggesting genetic specificity of lateralized gaze when viewing faces.

### Supplementary Information

Supplementary Information.

## Data Availability

The analyses presented here were preregistered (https://osf.io/kpqfx/). Deviations from the preregistration are discussed in the text. Custom-made scripts for pre-processing and statistical analyses will be made available upon reasonable request to corresponding author. Note that sharing of pseudonymized personal data will require a data sharing agreement, according to Swedish and EU law.
