# Supplementary material for: Genetic and environmental contributions to gaze lateralization across social and non-social stimuli in human infants
Source: Sci Rep. 2024 Feb 14;14:3668. doi: 10.1038/s41598-024-54373-6 (PMC10864339; doi:10.1038/s41598-024-54373-6)
Supplement: Supplementary file 1 — Supplementary Information. [file 41598_2024_54373_MOESM1_ESM.pdf]

Supplementary Information

Genetic and environmental contributions to gaze lateralization across social and non-social stimuli in human infants

Authors: Charlotte Viktorsson[1*], Ana Maria Portugal[1,2], Terje Falck-Ytter[1,2]

[1]Development and Neurodiversity Lab, Department of Psychology, Uppsala University; Uppsala, Sweden

[2]Center of Neurodevelopmental Disorders (KIND), Division of Neuropsychiatry, Department of Women's and Children's Health, Karolinska Institutet; Stockholm, Sweden

*Corresponding author. Email: charlotte.viktorsson@psyk.uu.se

Supplementary Information S1. Gaze lateralization for the lower part of the face.

When analyzing data from only the lower part of the face (**Figure 1**), a significant right gaze bias was found (mean = .391, $t(551) = -12.0$, $p < .001$, Cohen's $d = .51$).

There were no statistically significant associations between gaze lateralization at five months and concurrent general development ($\beta = -.05$, 95% CI: -.03; .14, $p = .226$), socio-communicative abilities at 14 months ($\beta = <-.01$, 95% CI: -.13; .09, $p = .707$), or language comprehension at 14 months ($\beta = .01$, 95% CI: -.09; .11, $p = .858$). Likewise, autistic traits at 36 months was not related to gaze lateralization ($\beta = <.01$, 95% CI: -.09; .12, $p = .792$) and neither was vocabulary at 36 months ($\beta = -.07$, 95% CI: -.19; .06, $p = .273$).

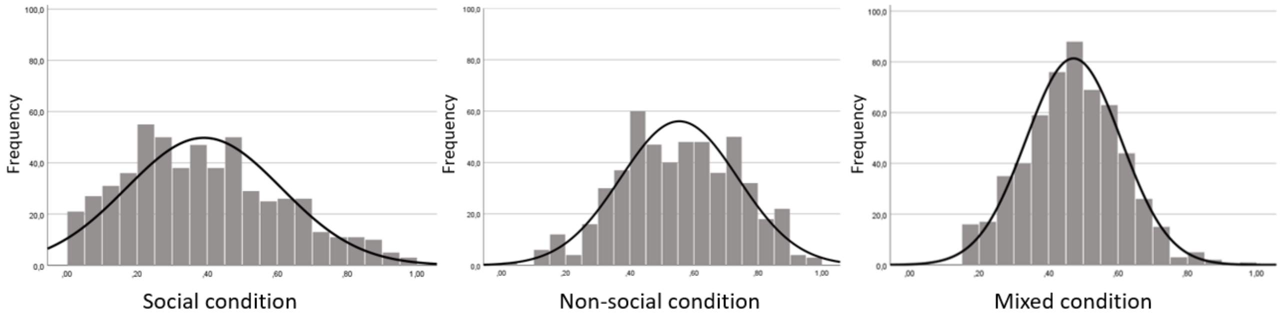

Figure S1. Distributional plots of the Social condition, Non-social condition, and Mixed condition.

Testing covariates and assumptions

**Social condition**					**Comparative fit with saturated model**		
**Model**	**-2LL**	**# parameters**	***df***	**AIC**	**Δ χ2**	**Δ df**	**p**
Fully sat.	-132.85	12	538	-108.86	-	-	-
Submodel 1	-131.71	10	540	-111.71	1.14	2	0.56
Submodel 2	-127.59	8	542	-111.59	5.27	4	0.26
Submodel 3	-126.84	7	543	-112.84	6.02	5	0.30
Submodel 4	-126.71	6	544	-114.71	6.15	6	0.41
Age	-131.86	11	539	-109.86	1.00	1	0.32
Sex	-132.54	11	539	-110.54	0.32	1	0.57

**Non-social condition**					**Comparative fit with saturated model**		
**Model**	**-2LL**	**# parameters**	***df***	**AIC**	**Δ χ2**	**Δ df**	**p**
Fully sat.	4415.20	12	500	4439.20	-	-	-
Submodel 1	4416.18	10	502	4436.18	0.98	2	0.61
Submodel 2	4418.56	8	504	4434.56	3.36	4	0.50
Submodel 3	4418.56	7	505	4432.56	3.36	5	0.64
Submodel 4	4421.46	6	506	4433.46	6.26	6	0.39
Age	4415.61	11	500	4437.61	0.40	1	0.53
Sex	4415.61	11	501	4437.61	0.41	1	0.52
**Mixed condition**					**Comparative fit with saturated model**		
**Model**	**-2LL**	**# parameters**	***df***	**AIC**	**Δ χ2**	**Δ df**	**p**
Fully sat.	-645.13	12	545	-621.13	-	-	-
Submodel 1	-644.29	10	547	-624.29	0.84	2	0.66
Submodel 2	-643.69	8	549	-627.69	1.45	4	0.84
Submodel 3	-643.69	7	550	-629.69	1.45	5	0.92
Submodel 4	-640.92	6	551	-628.92	4.21	6	0.65
Age	-643.93	11	546	-621.93	1.20	1	0.27
Sex	-644.19	11	546	-622.19	0.95	1	0.33

Table S1. The fully saturated model is the baseline model, which models the means and variances separately for each twin in a pair and across zygosity.
Submodel 1: Equating means across twins within a pair
Submodel 2: Equating means across zygosity
Submodel 3: Equating variances across twins within a pair
Submodel 4: Equating variances across zygosity
Age: Testing the significance of age
Sex: Testing the significance of sex
-2LL: Fit statistic, the lower the better fitting is the model
df: Degrees of freedom
AIC: An alternative fit index, lower value denotes better model fit
Δ χ2: Difference in −2LL statistic between two models, distributed χ2
Δ *df:* Difference in degrees of freedom between two models
